# Diagnosis, Management, and Prognosis of Cystic Fibrosis-Related Liver Disease in Children

**DOI:** 10.3390/diagnostics14050538

**Published:** 2024-03-03

**Authors:** Dana-Teodora Anton-Păduraru, Alice Nicoleta Azoicăi, Felicia Trofin, Alina Mariela Murgu, Dana Elena Mîndru, Ana Simona Bocec, Codruța Olimpiada Iliescu Halițchi, Gabriela Rusu Zota, Diana Păduraru, Eduard Vasile Nastase

**Affiliations:** 1 Department of Mother and Child Medicine, “Grigore T. Popa” University of Medicine and Pharmacy, 700115 Iaṣi, Romania; dana.anton@umfiasi.ro (D.-T.A.-P.); alice.azoicai@umfiasi.ro (A.N.A.); alina.murgu@umfiasi.ro (A.M.M.); mindru.dana@umfiasi.ro (D.E.M.); ana-simona.drochioi@umfiasi.ro (A.S.B.); olimpiada.iliescu@umfiasi.ro (C.O.I.H.); 2 “Sf. Maria” Children Emergency Hospital, 700309 Iasi, Romania; 3 Department of Preventive Medicine and Interdisciplinarity-Microbiology, “Grigore T. Popa” University of Medicine and Pharmacy, 700115 Iaṣi, Romania; 4 Department of Pharmacology, Clinical Pharmacology and Algesiology, “Grigore T. Popa” University of Medicine and Pharmacy, 700115 Iasi, Romania; rusu.i.gabriela@umfiasi.ro; 5 “Dr. C. I. Parhon” Clinical Hospital, 700503 Iaṣi, Romania; 6 Department of Internal Medicine II—Infectious Diseases, “Grigore T. Popa” University of Medicine and Pharmacy, 700115 Iasi, Romania; eduard-vasile.nastase@umfiasi.ro; 7 Clinical Hospital of Infectious Diseases “Sf. Parascheva”, 700116 Iasi, Romania

**Keywords:** cystic fibrosis, liver disease, children

## Abstract

Cystic fibrosis (CF) is a multifaceted disorder predominantly investigated for its pulmonary manifestations, yet patients with CF also exhibit a spectrum of extrapulmonary manifestations, notably those involving the hepatobiliary system. The latter constitutes the third leading cause of morbidity and mortality in individuals with CF. Cystic fibrosis-related liver disease (CFLD), with an escalating prevalence, manifests diverse clinical presentations ranging from hepatomegaly to cirrhosis and hepatopulmonary syndrome. Consequently, early detection and appropriate management are imperative for sustaining the health and influencing the quality of life of CF patients afflicted with CFLD. This review aims to consolidate existing knowledge by providing a comprehensive overview of hepatobiliary manifestations associated with CF. It delineates the clinical hepatobiliary manifestations, diagnostic methodologies, incorporating minimally invasive markers, and therapeutic approaches, encompassing the impact of novel CFTR modulators on CFLD. Given the exigency of early diagnosis and the intricate management of CFLD, a multidisciplinary team approach is essential to optimize care and enhance the quality of life for this subset of patients. In conclusion, recognizing CF as more than solely a pulmonary ailment, the authors underscore the imperative for further clinical investigations to establish a more robust evidence base for CFLD management within the continuum of this chronic disease.

## 1. Introduction

Cystic fibrosis (CF) is a multifaceted disorder impacting various organs, excluding the brain, and stands as the most prevalent monogenic autosomal recessive disease within the Caucasian population. Its chronic, progressive, and potentially fatal nature is characterized by a pervasive involvement of exocrine glands, resulting in the clinical triad of exocrine pancreatic insufficiency, chronic lung diseases, and elevated chloride and sodium concentrations in perspiration [1]. The cystic fibrosis transmembrane conductance regulator (CFTR) protein, comprising 1480 amino acids and identified in 1989, is encoded by the CF gene, with the F508 del mutation being the most prevalent in Europe and North America among over 2000 documented mutations [2].

While CF is predominantly acknowledged for its extensively studied pulmonary manifestations, patients often concurrently exhibit hepatobiliary complications due to the robust expression of the CFTR protein in cholangiocytes [3]. Cystic fibrosis-related liver disease (CFLD) has emerged as a significant complication, and impacts morbidity and mortality and has become the third leading cause of adverse outcomes in CF patients, following respiratory diseases and pulmonary transplant complications [4,5,6,7]. The initial recognition of hepatobiliary involvement in CF dates back to Anderson in 1938, with subsequent elucidation of biliary cirrhosis by Farber & Bodian [8]. Clinical manifestations encompass hepatomegaly, cholestasis signs, steatosis, gallbladder abnormalities, cirrhosis, hepatopulmonary syndrome, and portal hypertension, all of which profoundly impact quality of life and long-term prognosis. Advances in CFLD diagnosis, facilitated by the introduction of minimally invasive markers for liver fibrosis, enable early detection [9].

Furthermore, the advent of novel CFTR modulator therapies holds the potential to alter the course of CFLD evolution. However, limitations include the constrained scope of studies on the extrapulmonary effects of these modulators, eligibility constraints for certain CFLD patient subgroups, and concerns regarding hepatotoxicity restricting their application. Hence, the recognition and management of CFLD within an interprofessional team setting are pivotal for preserving the health of CF patients and enhancing their overall quality of life.

The primary aims of this study were to amalgamate existing knowledge on CFLD, offering comprehensive insights into its epidemiology, pathophysiology, clinical manifestations, and diagnostic modalities. Additionally, the investigation encompassed an evaluation of treatment strategies, with a particular focus on novel therapeutic interventions designed to address the fundamental impact of CF and their subsequent influence on the hepatobiliary tract.

## 2. Methods and Search Strategy

This systematic review encompasses a compilation of 112 studies. The meticulous selection and curation of articles for inclusion adhered to a stringent set of criteria, centered around the pivotal question: “What are the diagnostic methods and management possibilities for cystic fibrosis-related liver disease in children?” These criteria encompassed alignment with research objectives, year of publication, scientific research categorization, and an assessment of the quality of the results presentation.

The initial identification of records involved database searches utilizing specified terms such as “cystic fibrosis”, “cystic fibrosis-related liver disease”, and “children” on reputable platforms, namely PubMed, Google Scholar, and Cochrane databases. Subsequent to the initial database searches, a comprehensive screening process was initiated, commencing with the removal of duplicate records. Following this, an evaluation of article titles was conducted to exclude those lacking relevance to the study. Articles exhibiting promise based on title and abstract underwent scrutiny of their full text. Additionally, supplementary references meeting the defined criteria were identified through a manual search within the reference lists of retrieved articles.

**Figure 1:**
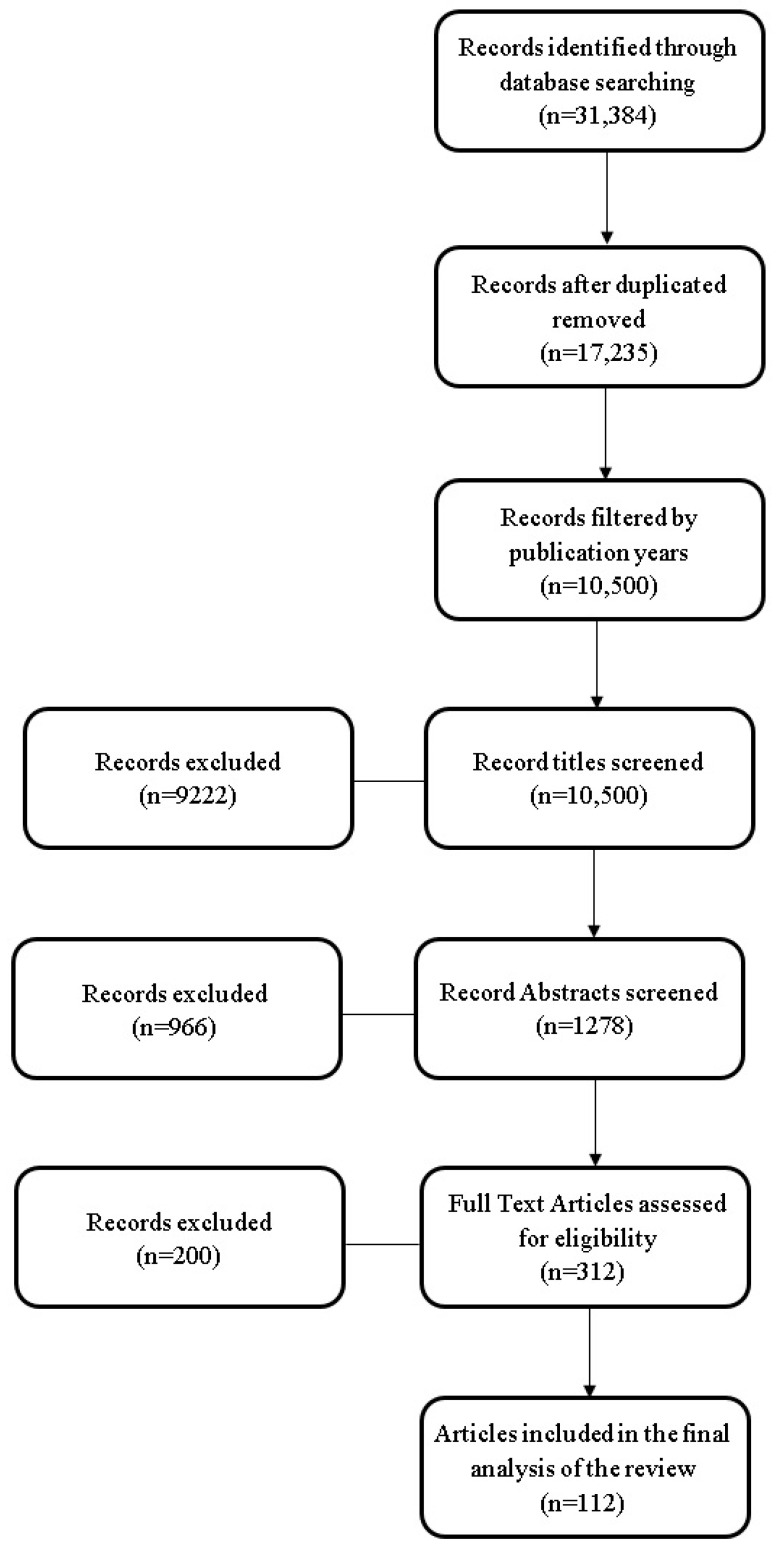
The review flowchart.

The results from all included articles in the references were synthesized and structured systematically to form the foundation of this review article. A structured framework was methodically constructed to organize relevant data. The accompanying flowchart (Figure 1) delineates the sequential progression of information throughout the review process, providing a visual representation of the records identified, incorporated, and excluded.

## 3. Results

### 3.1. General Data

#### 3.1.1. Definition

In accordance with the criteria proposed by Debray et al. (2011) and endorsed by the European Cystic Fibrosis Society (EFCS) and Cystic Fibrosis Foundation (CFF), the diagnosis of CFLD is established when two of the following factors are concurrently present: hepatomegaly and/or splenomegaly, liver biochemical changes including transaminase (ALT, AST) and gamma-glutamyl transpeptidase (GGT) elevations exceeding 1.5–2 times the normal values persisting for over 6 months, alterations in liver ultrasounds indicative of coarseness, nodularity, increased echogenicity, and signs of portal hypertension, and abnormal liver biopsy results depicting focal biliary/multilobular cirrhosis [10,11].

Alternatively, the novel criteria introduced by Koh et al. (2017) incorporate non-invasive biomarkers, including an AST/ALT ratio of ≥1, a fibrosis index of ≥3.25, and an aspartate aminotransferase (AST)/platelet ratio (APRI) exceeding 0.50 [12]. Comparative analyses with the Debray et al. (2011) criteria reveal a 17.70% increase in CFLD identification through the application of the new criteria [5,6]. Attempts to integrate an ultrasound scoring system for CF patients into the definition criteria, although made by some authors, lacked validation and incorporation [13].

#### 3.1.2. Epidemiology

The prevalence of CFLD exhibits a considerable range, spanning from 2% to 62%, with an observed age-dependent escalation from 3.7% at 5 years to 32.2% at 30 years [5]. A study by Boelle et al. (2019) denotes a 1% annual increase in CFLD incidence past the age of 5 [14]. Leung et al. (2023) report that approximately 10% of newborns during the neonatal period can develop cholestatic liver disease, with 50% of CF patients exhibiting transiently elevated transaminase values in the initial two years of life [11]. Hepatobiliary damage is evident before or during puberty in 15–17% of cases, with an earlier onset in individuals with a history of meconial ileus [15,16]. Notably, 5% of patients below the age of 13 present nodularity indicative of non-cirrhotic portal hypertension or cirrhosis [11].

Primary risk factors for CFLD development include male gender, a history of meconium ileus (with a fivefold higher risk), pancreatic insufficiency, particularly in patients with class I, II, or III mutations, CF-related diabetes (CFRD), and the SERPINA-1 allele [15,17,18,19,20]. Additional implicated factors encompass chronic infections, malnutrition, certain drugs, histocompatibility complex antigens, and intestinal dysbiosis [19]. Mild mutations (class IV and V) are infrequently observed in CFLD patients [21]. The manifestation of liver disease can be influenced by genetic modifiers, including the protease inhibitor gene, mannose-binding lectin 2 gene, beta-TGF gene, and glutathione S-transferase 1 gene [16]. Liver disease induced by long-term total parenteral nutrition is noted in 40–60% of patients. The onset of liver disease in females before puberty is attributed to endocrine factors, particularly estrogens, while environmental factors demonstrate a negligible influence [2,5,15,18,22,23,24,25].

#### 3.1.3. Pathophysiology of CFLD

The etiological mechanisms underlying liver disease in the context of CF remain incompletely elucidated, although certain CFTR gene mutations, particularly the F508 del homozygous mutation, are identified as augmenting the risk and severity of manifestation [2]. Within the liver, CFTR is expressed on the luminal membrane of cholangiocytes, constituting the biliary tree cells. The biliary epithelium, forming a 3D tubular structure in the liver, functions to collect primary bile from hepatic canaliculi at the Hering canals and transport it to the duodenum. Bile undergoes modifications during this transit to align with digestive requirements. Cholangiocytes, acting as transporters, regulate bile fluidity and alkalinity. The duodenum-secreted secretin binds to its receptor on the basolateral membrane of cholangiocytes, leading to an increase in intracellular cyclic AMP. This activation prompts protein kinase A-mediated chloride efflux through CFTR into the biliary lumen. Simultaneously, an electrolytic gradient facilitates chlorine reabsorption into cells, while anion-exchange protein 2 mediates bicarbonate secretion into the lumen. This gradient further supports luminal water movements through aquaporins, specialized water channels. Considering CFTR as a pivotal determinant of bile secretion, CFLD is conceptualized as a “channelopathy” [2]. CFTR dysfunction perturbs cholangiocyte function, resulting in alterations in bile composition, including changes in hydration and alkalinity, abnormal bile transport, and modifications in bile acids. The gut–liver axis assumes critical importance in bile acid homeostasis, and CFLD arises from the retention of toxic acids (cholic acid, chenodeoxycholic acid, taurocholic acid), initiating an inflammatory response and causing epithelial damage [15,18,20,25,26,27,28]. Mutations in the CFTR gene result in the accumulation of hyperviscous bile in the biliary tree, leading to hepatocyte and cholangiocyte damage, inflammation, focal fibrosis, and progression to multinodular cirrhosis, portal hypertension, and liver decompensation. Prolonged cholestasis renders the biliary epithelium susceptible to cytotoxic destruction by inflammatory mediators such as cytokines and chemokines.

The concept of the gut–liver axis delineates a bidirectional relationship between the gut, its microbiota, and the liver, arising from the integration of signals from dietary, genetic, and environmental factors. Facilitated by the portal vein, this interaction allows the transport of gut-derived products directly to the liver and reciprocally involves the liver’s feedback route of bile and antibody secretion to the intestine [29,30]. Metabolites produced by the gut microbiome establish connections with the liver through systemic circulation, portal circulation, and the bile duct. These metabolites impact immunity, metabolism, and bile acid production, while bile acids produced in the liver regulate gut microbial composition and gut epithelial barrier integrity. The consequences encompass increased intestinal permeability and the translocation of microorganisms into the portal and liver systems, leading to inflammatory responses [30].

CFTR dysfunction exerts an impact on the microbiome, inducing dysbiosis characterized by prolonged transit time and bacterial overpopulation, thereby prompting intestinal inflammation, the proliferation of pathogenic bacteria, and their translocation into the portal circulation. This disruption in the microbial equilibrium within the intestine is implicated in diverse liver diseases, including CF, contributing to heightened intestinal permeability. The consequent ingress of inflammatory mediators into the portal circulation triggers the activation of hepatic stellate cells [19].

**Figure 2:**
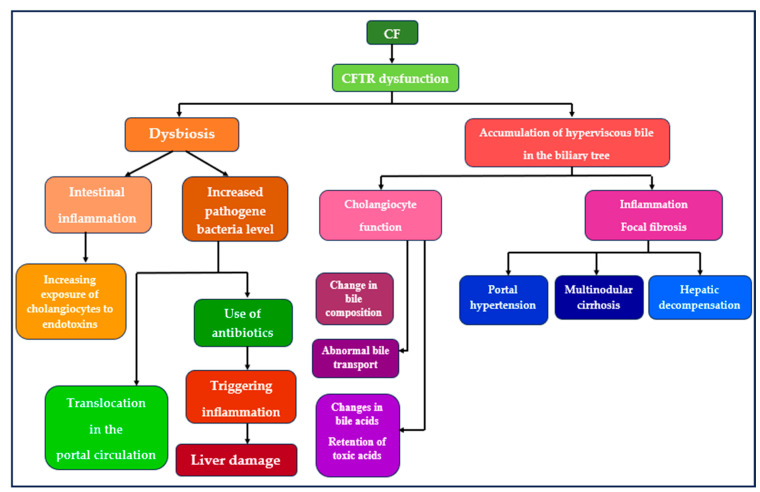
Pathophysiology of CFLD.

Numerous studies have underscored the perturbation of the intestinal microbiome in cirrhosis, with prevailing species such as *Streptococcus*, *Veilonella*, *Megasphaera*, *Dialister*, *Atopodium*, and *Prevotella* being identified [31,32,33]. Findings from the preclinical investigation by Rager et al. (2023) demonstrating a reduced abundance of *Bacteroides* and an increased abundance of *Clostridium* lend support to the role of gut dysbiosis in the pathogenesis of CFLD [34]. The research conducted by Debray et al. (2018) on mice concluded that CFTR deficiency, leading to abnormal intestinal permeability, coupled with dysbiosis induced by diet and immune-related genetic susceptibility, could potentially foster CF-related cholangiopathy. Examination of the fecal microbiota of CFTR −/− mice exhibiting CF-related cholangiopathy revealed discernible differences in species abundance, with an elevated proportion of Escherichia coli [27]. Alterations in the diversity and composition of the gut microbiota in CF patients, characterized by an increase in intestinal microbial alpha diversity, may play a role in the development of CFLD [15,18,25,26,28,34]—refer to Figure 2.

Altered microbiota also leads to the disruption of bile acid homeostasis by impairing the activation pathway of the farnesoid X receptor (FXR) and fibroblast growth factor 19 (FGF19) [35]. The disruption of bile acid homeostasis and the translocation of bacterial endotoxins and pathogen-associated molecular patterns (PAMPs) into the portal venous system and liver can trigger uncontrolled biliary inflammation by activating the Src/NF-κB signaling pathway in cholangiocytes with altered CFTR. Moreover, in vivo studies on mice with CFTR deficiency have demonstrated a defect in the function of the peroxisome proliferator-activated receptor gamma (PPARγ), whose stimulation could attenuate NF-κB-mediated inflammation [36]. Additionally, in individuals with CF and liver disease, a reduction in ileal expression of fibroblast growth factor 15 has been observed, potentially contributing to biliary toxicity [22]. Factors contributing to the precipitation of bile acids in the bile ducts include reduced synthesis of bile acid salts, diminished absorption of bile acids from the lumen of the small intestine, and altered bile flow from the liver into the duodenal lumen [25].

Abnormal bile content, increased excretion of bile acids in fecal matter, and the formation of lithogenic bile, where bile acids are mixed with glycine, play a role in the pathogenesis of gallstones. No correlation has been observed between gallstone formation and the administration of pancreatic enzymes [25]. The chronic use of antibiotics for the treatment of infections caused by various pathogens can trigger an inflammatory cascade leading to hepatic impairment—refer to Figure 2 [37].

### 3.2. Diagnosis

#### 3.2.1. Clinical Data

From a clinical perspective, the manifestation of the disease exhibits considerable variability. The hepatic phenotype among individuals sharing the same genotype is diverse, suggesting the involvement of both environmental factors and genetic modifiers [10]. No correlations have been identified between liver lesions and lung lesions, respiratory insufficiency, the degree of malnutrition, meconium ileus, and distal intestinal obstruction syndrome (DIOS). Conversely, malnutrition can influence liver function in patients with CF [25]. Certain patients may remain asymptomatic, displaying solely elevated liver test values (LTs), while approximately 5–10% develop multilobular cirrhosis within the initial decade of life [2,26].

- CFLD and meconium ileus

In infants, liver damage may manifest as cholestasis, often coexisting with meconium ileus. Approximately 15–20% of newborns experience meconium ileus more frequently among monozygotic twins and those with a familial history of the condition. In the initial 24–48 h of life, affected individuals exhibit abdominal distension, vomiting, and an inability to eliminate meconium [38]. Additional presentations include hepatomegaly (commonly resulting from hepatic fatty infiltration or focal biliary fibrosis), alterations in stool appearance and color, abdominal cramps, early satiety due to organomegaly, variceal hemorrhage, gall microvesicles, and persistent elevation in liver enzymes. Typically, an enlarged left lobe protruding centrally is associated with splenomegaly, leading to abdominal pain or discomfort [5,10,11,15].

B. Liver disease without portal hypertension

Steatosis, present in approximately 60% of patients, especially those with malnutrition, excessive fat consumption, impaired phospholipid metabolism, and essential fatty acid deficiency, is not directly linked to the CFTR defect [11]. Peripheral signs of chronic liver disease include nevi, palmar erythema, jaundice, edema, and distension of veins on the abdominal wall [10].

C. Liver disease with cirrhosis/portal hypertension

Focal biliary cirrhosis, stemming from obstruction of the intrahepatic bile ducts and contributing to prolonged neonatal jaundice, exhibits an increased prevalence with advancing age. Despite receiving adequate nutrition, fatty liver infiltration is observed in 30–70% of patients [38]. This condition stands as the pathognomonic lesion in CF, distinguished by focal portal fibrosis and cholestasis, occasionally serving as a precursor to multilobular cirrhosis. Multilobular cirrhosis, typically infrequent (prevalence of 5.6%), is characterized by the presence of multiple regenerative nodules with diffuse involvement of the liver parenchyma [39,40]. Hepatic encephalopathy is an uncommon occurrence in CF cirrhosis, typically arising from infections, excessive protein intake, gastrointestinal bleeding, or post-application of portosystemic shunts for managing portal hypertension [15,41]. Others may present with decompensated liver disease characterized by portal hypertension [2,26].

D. Hepatopulmonary syndrome

Hepatopulmonary syndrome manifests as progressive hypoxemia devoid of an identifiable respiratory etiology, necessitating the measurement of oxygen saturation in both the supine and upright positions. A significant reduction in saturation (5%) upon assuming an upright posture is indicative of hepatopulmonary syndrome [41]. Concurrent colonization with *Aspergillus fumigatus* and *Stenotrophomonas maltophilia* has been noted in association with liver disease [22]. Boëlle et al.’s study (2018) revealed that severe CFLD patients had a slightly worse forced expiratory volume in the first second (FEV1) compared to those without liver damage, and CF patients exhibited altered nutritional status compared to the general population, particularly those with severe CFLD [14].

E. Gallbladder and biliary tract involvement

Anomalies in both intra- and extrahepatic bile ducts, such as strictures, dilations, and calculi, resembling those observed in sclerosing cholangitis, have been documented [42]. Several studies have indicated that healthy carriers exhibit a twofold higher risk of bile duct obstruction [43]. Narrowing of the distal regions of bile ducts is prevalent, occurring in approximately 90% of patients and contributing to gallstone formation. Vesicular hydrops and gallstones are more frequent in CF patients compared to the general population, with cholelithiasis found in 14–24% of cases [25].

Gallbladder abnormalities are identified in 24–50% of cases and encompass microvesicles resulting from developmental abnormalities of the fetal gallbladder, atresia, or stenosis leading to gallbladder atrophy. Treatment is generally not required. Other abnormalities include gallstones, choledocholithiasis, and intrahepatic stones due to the loss of bile acids (BAs) in stools, resulting in the formation of lithogenic bile. Additionally, cholecystitis may arise from biliary obstruction caused by stones or sludge [11,39].

F. CFLD and association with endocrine diseases

Individuals with CFLD face an elevated risk of developing CF-associated bone disease, hypogonadism, as well as experiencing vitamin deficiencies and endocrinopathies, including cystic fibrosis-related diabetes (CFRD) [16,43,44]. Conversely, the insulin resistance observed in patients with CFRD contributes to increased fibrosis and the development of cirrhosis in classic CFLD cases [45].

G. The occurrence of Distal Intestinal Obstructive Syndrome (DIOS) in CFLD post-transplant children

Morton et al. (2009) reported DIOS in 10.7% of long-term transplant recipients, with some having a prior history of meconium ileus [46]. Similarly, Gilljam et al. (2003) observed DIOS in 20% of their Canadian study cohort, with one-third experiencing recurrent episodes [47]. Valamparampil et al. (2021) corroborated these findings, indicating that over 20% of transplanted patients face this potentially life-threatening complication. Furthermore, patients with a history of DIOS episodes or abdominal surgeries within five years prior to lung transplantation are at heightened risk for post-transplant DIOS [20]. Dowman et al. (2011) noted that while 10.5% of patients with a meconium ileus history did not develop post-transplant DIOS, 21% without such history did [48]. Given this elevated risk, diligent post-operative monitoring of these patients is strongly advised [47].

#### 3.2.2. Laboratory and Paraclinical Findings

Specific diagnostic tests for CFLD are currently unavailable, contributing to potential delays in diagnosis, particularly in cases with advanced liver damage [23].

*A*. Laboratory Tests

Elevated transaminase levels (ALT, AST) exceeding twice the normal range for at least three months signify advanced liver damage. However, these values demonstrate low specificity and sensitivity. In some instances, non-specific biochemical anomalies present in over 50% of infants may normalize within 2–3 years without impacting subsequent liver disease development [16,49]. Isolated elevation of transaminases with a normal GGT value indicates the presence of steatosis [25]. The persistence of elevated GGT values is associated with the onset of CFLD within two years, according to Bodewes et al. (2015), or the development of cirrhosis, as noted by Woodruff et al. (2017) [50,51]. A decrease in alkaline phosphatase (ALP) levels may suggest improved CFTR function in cholangiocytes. Liver failure should be considered if prothrombin time and coagulation factors II, VII, and X remain low despite vitamin K supplementation [10]. Persistent hypoglycemia below 70 mg/dL may raise suspicion of liver dysfunction [41]. Lower levels of vitamins A and E are correlated with worsening liver function and an increased rate of pulmonary exacerbations [25].

*B*. Liver Fibrosis Index

Liver fibrosis indices are employed to detect advanced fibrosis and portal hypertension, offering potentially higher specificity compared to laboratory tests:

- The GGT-to-platelet ratio (GPR) serves as a predictor of CFLD, with a value between 0.20 and 0.32 associated with moderate hepatic fibrosis, while a value exceeding 0.68, coupled with a heterogeneous liver appearance on ultrasound, predicts CFLD risk [11].
- The albumin–bilirubin score (ALBI) proves superior to the Child–Pugh score in assessing liver disease severity in CF patients, according to Poetter-Lang et al.’s study (2019) [52].
- The AST-to-platelet ratio index (APRI) is considered a reliable marker for hepatic fibrosis and is recommended for annual assessment. A value of ≥0.5 necessitates further imaging evaluation, while a value ≥ 1 warrants additional investigations, including biopsy.
- The Fibrosis-4 index (FIB-4).
- Other pertinent parameters in liver fibrosis diagnosis include aminopeptides type III procollagen, collagen I and IV, laminin, hyaluronic acid, cytokines, and chemokines [25].

*C*. Paraclinical Investigations

(a) Liver Ultrasound

Liver ultrasound is instrumental in identifying hepatic steatosis, focal biliary fibrosis, multilobular cirrhosis, ascites, bile duct damage, and splenomegaly. Echo-Doppler examination may reveal cirrhosis-related findings such as nodules, steatosis, parenchymal heterogeneity, increased periportal echogenicity, reversal of blood flow in the portal vein or recanalized umbilical vein, enlarged collateral veins, gastro-esophageal varices, and signs of portal hypertension (splenomegaly, ascites, mesenteric edema) [41]. Siegel et al. (2021) propose that a heterogeneous echogenic aspect characterized by a nodular liver pattern, with or without portal hypertension, could serve as a predictive biomarker for the risk of developing CFLD in children [53]. Elevated fibrosis indices correlate with nodular liver appearance on ultrasound, the presence of portal hypertension, varices, and advancing fibrosis [54].

(b) Computer Tomography (CT) and Magnetic Resonance Imaging (MRI)

Gadoxetic acid-enhanced MRI, as observed by Poetter-Lang et al. (2019), can diagnose CFLD in its early stages based on three independent imaging features: altered gallbladder morphology, periportal tracking, and periportal fat deposition [52]. CT and MRI are valuable tools for distinguishing between fibrosis and steatosis and for detecting bile duct abnormalities [15].

(c) Elastography

Elastography plays a crucial role in distinguishing fibrosis stages 1–2 from stages 3–4, influencing prognosis and treatment decisions. “Shear wave” or “vibration-controlled transient” elastography, a non-invasive technique, proves useful in diagnosing liver disease by quantifying fibrosis and steatosis, and measuring liver stiffness [11,25,55]. Gominon et al. (2018) observed that the slope of worsening liver stiffness is greater in patients who will develop CFLD, suggesting the need for annual transient elastography to detect the risk of severe liver disease at an earlier stage [56]. Combining serum-based tests (FIB-4, non-alcoholic fatty liver disease fibrosis score) with elastography techniques enhances diagnostic accuracy and serves as screening and confirmatory testing [57].

Lewindon et al. (2019) propose that liver stiffness measurements (LSMs) by transient elastography, combined with AST-to-platelet ratio index (APRI), improve diagnostic accuracy and differentiate children with CF with mild–moderate fibrosis from those with advanced fibrosis. According to the research by Lewindon et al. (2019), LSMs exhibited a notable increase in children with CFLD, reaching 10.7 ± 2.4 kPa. They suggested a cutoff value of 5.55 kPa for CFLD patients. Additionally, in their study, a cutoff value of 8.7 kPa effectively distinguished patients with stage F3–F4 fibrosis from those with stage F1–F2 fibrosis [58].

The literature mentions varying LSM cut-off values for CFLD detection [59,60,61,62], ranging from 5.3 kPa [63] to 7.1 kPa [64]. Wiecek et al. (2022) introduced a lesion scale assessment in liver elastography based on both the stage of fibrosis and the LSM. Their assessment is presented in the following Table 1 [65].

Højte et al. (2020) suggest the combination of ultrasound examination with FibroScan or shear wave elastography, along with GGT levels, as diagnostic markers for CFLD [66]. Despite recent advancements in techniques like transient elastography, their ability to distinguish various phenotypical presentations or predict progression to clinically relevant cirrhosis or portal hypertension remains unestablished [67].

(d) Liver Scintigraphy

Hepatic scintigraphy serves to unveil compromised biliary drainage, intra- and extrahepatic bile duct dilation, and delayed biliary excretion, providing a means to monitor the response to treatment with ursodeoxycholic acid (UDCA) [10,16].

(e) Endoscopic Retrograde Cholangiography (ERCP)

ERCP, being an invasive procedure carrying potential complications, is exclusively recommended for patients with sclerosing cholangitis and choledocholithiasis [16]. Magnetic resonance cholangiography has revealed bile duct abnormalities even in patients without evident liver damage [10].

(f) Liver Biopsy

Microscopic examination of liver biopsy specimens, considered the “gold standard” for diagnosing and staging liver disease, highlights acinar steatosis, cholestasis, bile duct proliferations, portal inflammation, varying degrees of fibrosis, and regenerative nodular hyperplasia. However, due to the focal distribution of fibrosis, liver biopsy may not be a marker of absolute accuracy in CFLD [15,68]. Some authors question its adequacy for establishing a diagnosis, recommending the use of criteria established by Debray et al. (2011) [10]. Moreover, the invasive nature of liver biopsy poses a risk of under- or overestimating lesions, which can be mitigated by a dual-pass biopsy [69].

(g) Esophagogastroduodenoscopy (EGD)

After diagnosing CFLD, the primary objective of management is to mitigate the complications associated with portal hypertension and cirrhosis. Portal hypertension commonly accompanies advanced liver disease and can lead to severe complications, such as hemorrhage from esophageal and gastrointestinal varices. Although specific guidelines regarding the prevention and management of acute variceal bleeding due to portal hypertension in CFLD patients are lacking, it is recommended that all CFLD patients with cirrhosis undergo screening for esophageal varices using EGD [70,71].

There is currently no consensus on the optimal timing for endoscopic screening for esophageal varices. Additionally, approximately 2.5% of pediatric patients undergoing upper endoscopy experience serious complications. Therefore, there is a demand for non-invasive/non-endoscopic tests to detect the presence of varices, particularly in the pediatric population [72]. If large esophageal varices are detected during the EGD examination, variceal prophylaxis is necessary, which may involve pharmacologic, endoscopic, surgical, or interventional radiology interventions [70].

#### 3.2.3. Differential Diagnosis

Persistent elevation of transaminase values necessitates a thorough differential diagnosis to distinguish the condition from other diseases, including but not limited to viral hepatitis, α1-antitrypsin deficiency, autoimmune hepatitis, celiac disease, Wilson’s disease, hemochromatosis, essential fatty acid and carnitine deficiencies, drug-induced liver injury, and metabolic diseases such as non-alcoholic fatty liver disease (NAFLD) [15,41]. Additionally, the following conditions should be considered in the differential diagnosis:

- Non-alcoholic fatty liver disease, where hepatic steatosis is the predominant hepatic manifestation, observed in 20–60% of cases;
- Primary sclerosing cholangitis, characterized by inflammation and fibrosis of the intra- and extrahepatic bile ducts, presenting similar histological and radiological features;
- Secondary sclerosing cholangitis, exhibiting morphological similarities to the primary form, but recurrent pancreatitis may be deemed a causative factor for secondary cholangitis [11,15].

### 3.3. CFLD Management

#### 3.3.1. Therapeutic Approaches

No definitive treatment has been identified for effectively retarding the progression of liver disease in cystic fibrosis. The primary objective of treatment is to impede liver damage and mitigate associated complications such as cirrhosis and portal hypertension. Achieving this goal necessitates the collaboration of a multidisciplinary team comprising a pediatrician, hepatologist, gastroenterologist, nutritionist, radiologist, and surgeon [24].

A. Nutritional Interventions

Nutritional support is directed towards ensuring elevated protein and lipid intake, along with the administration of fat-soluble vitamins. For individuals with cystic fibrosis-related liver disease (CFLD), the following nutritional recommendations are advised:

- Increase daily food intake by 150%, emphasizing a higher percentage of fats, occasionally supplemented with carbohydrates (glucose polymers) cautiously due to the risk of cystic fibrosis-related diabetes (CFRD).
- Allocate 40–50% of caloric intake to fats, supplemented with medium-chain triglycerides (MCT) and polyunsaturated fatty acids (PUFAs). Research on CFTR+/+ and CFTR−/− mice demonstrated that a diet rich in MCT facilitated gallbladder emptying without significant postprandial differences in gallbladder volumes between the two groups of mice [27].
- Consume proteins at a rate of 3 g/kg body weight/day for those without indications of liver failure.
- Administer fat-soluble vitamins as supplements: vitamin A at 5000–15,000 IU/day, vitamin E at 100–500 mg alpha-tocopherol/day, vitamin D at 50 ng/kg body weight/day (up to 1 μg), and vitamin K at 1–10 mg/day, with regular plasma level monitoring to prevent deficiency or toxicity.
- Abstain from alcohol [10,11].

Salt supplementation is discouraged for patients with cirrhosis and portal hypertension due to the associated risk of developing ascites. In cases of anorexia, enteral feeding through a nasogastric tube is recommended. Gastrostomy is not advisable for individuals with CF and advanced liver disease featuring varices and portal gastropathy, primarily due to the risk of gastric bleeding [10].

B. Pharmacological Interventions

Ursodeoxycholic acid (UDCA) is a hydrophilic, non-toxic bile acid possessing cytoprotective, antiapoptotic, choleretic, and immunomodulatory properties. UDCA facilitates chloride secretion through calcium-dependent chloride channels, diminishes cholic acid concentration in bile, reduces mucin secretion, mitigates the toxic effects of hydrophobic bile acids, and enhances biochemical and histopathological parameters and essential fatty acid homeostasis [25,28]. In a study by Shimokura et al. (1995), pharmacological concentrations of UDCA were found to elevate intracellular calcium levels, induce chloride secretion, and augment bile flow through direct stimulation of ductular secretion [73]. The recommended treatment for CFLD involves administering UDCA at a dosage of 20–30 mg/kg body weight/day, distributed in at least two doses to enhance absorption. However, the efficacy of this treatment remains a subject of uncertainty [2,5,10,15,16,18,22,74]—Table 2.

UDCA is recommended for administration before the onset of severe liver damage to forestall disease progression and ameliorate fibrosis levels [16,25]. In cases of children exhibiting cholestasis and meconium ileus, a prescribed regimen entails a 2–3 month course of 10–20 mg/kg body weight/day. Assessments for cholestasis and cytolysis are conducted at 3 and 6 months following the commencement of UDCA treatment, with necessary dose adjustments if required [10]. It is essential to note that substantial UDCA doses may elicit adverse effects, as evidenced by the occurrence of colonic adenocarcinoma in adults [41]. Moreover, caution is warranted, as elevated UDCA doses have demonstrated deleterious effects in patients with primary sclerosing cholangitis due to its transformation into lithocholic acid in the colon—a hydrophobic bile acid with potential toxicity [81,82]. Post-transplantation, UDCA usage has shown associations with enhanced aminotransferases, normalized bile composition, and improved liver stiffness [75,78]. Future investigations should explore the safety and efficacy of bile analogs or derivatives such as obeticholic acid and norursodeoxycholic acid [41].

Administration of short-chain fatty acids (SCFAs) to individuals with hepatic steatosis and insulin resistance has demonstrated beneficial effects [30].

The presence of esophageal varices necessitates pharmacological (octreotide), endoscopic (endoscopic variceal band ligation, band ligation +/− sclerotherapy), surgical (portosystemic shunting), or interventional radiology interventions. Refractory bleeding may warrant a transjugular intrahepatic portosystemic shunt (TIPS). The use of non-selective beta-blockers (propranolol) is contraindicated due to bronchoconstriction. Analogues of somatostatin or vasopressin are recommended to diminish splanchnic flow [15,16,20,41]. Thrombocytopenia and leukopenia resulting from hypersplenism generally do not require treatment [10]. The presence of ascites signifies an unfavorable prognosis, necessitating fluid and sodium restriction alongside diuretic administration (spironolactone, furosemide). Treatment and monitoring of gallbladder disease align with protocols for patients without CF [11]. Due to its antioxidant properties, coenzyme Q10 can be recommended for CFLD patients. Additionally, taurine, in a dose of 30 mg/kg body weight/day, has demonstrated favorable effects in severe liver failure cases [8]. Anti-hepatitis A and B vaccination is recommended as a preventive measure [10,15,22]. It is crucial to exclude hepatotoxic medications such as non-steroidal anti-inflammatory drugs (NSAIDs) and salicylic acid due to the associated risk of bleeding [11].

C. CFLD and CFTR Modulators

(a) Impact of CFTR Modulators on CFLD

The introduction of CFTR modulator therapies designed to correct and potentiate CFTR in cholangiocytes, endothelial cells, and platelets aims to impede or arrest the progression of CFLD [69]. While clinical studies on the effects of CFTR modulators on pulmonary function are abundant, investigations into their impact on the extrapulmonary effects, particularly liver damage in CF, are still limited, leaving uncertainties regarding their influence on the advancement of liver disease—Table 3.

(b) Hepatotoxicity Associated with CFTR Modulators

The commencement of CFTR modulator therapy necessitates normal liver function. Elevations in AST and ALT exceeding five times the upper limit of normal or an increase greater than three times in conjunction with a bilirubin rise exceeding twice the normal level necessitate discontinuation of the treatment [8].

No adjustment in dosage is recommended for patients with mild hepatic impairment (Child–Pugh class A). In cases of moderate hepatic insufficiency (Child–Pugh class B), treatment is not recommended; however, if administered, the dosage should be reduced. Patients with severe liver failure (Child–Pugh class C) should refrain from CFTR modulator treatment, given the absence of clinical studies [93].

D. The Role of Probiotics in CFLD

Supplementation with prebiotics that foster the proliferation of beneficial bacteria can impact gastrointestinal and respiratory microflora, influencing intestinal inflammation and exacerbations [94]. These bacteria play a role in host metabolism, affecting bile acid metabolism, short-chain fatty acid (SCFA) production, and reducing plasma lipopolysaccharide concentration. Gut microbiota deconjugate bile acids (BAs) through the activity of microbial enzymes produced by various microbial species (*Lactobacillus*, *Bifidobacterium*, *Enterococcus*, *Clostridium* genera). *Lactobacillus plantarum*, in particular, may contribute to pathogen elimination and the reduction of dysbiosis [95,96].

E. Surgical Management of CFLD

Variceal ligation does not eliminate the risk of rebleeding; in such cases, the recommended course of action is the transjugular placement of an intrahepatic portosystemic stent. In refractory cases, an alternative is the surgically placed portosystemic shunt, albeit with associated risks of complications such as encephalopathy and acute liver failure [15,16,41]. Portosystemic shunt procedures can be contemplated as an alternative to liver transplantation, with transjugular intrahepatic portosystemic shunts (TIPSs) serving as a bridge to liver transplantation [69]. Sclerotherapy is not recommended as a primary prophylaxis measure due to the risk of bleeding during and after the procedure [10].

F. Liver Transplantation

Liver transplantation, recommended for individuals with CFLD, substantially enhances survival compared to non-transplanted counterparts [97]. Indications for liver transplantation include the following:

- Liver dysfunction or advanced portal hypertension;
- Progressive liver failure marked by hypoalbuminemia (below 3 g/dL), coagulopathy (INR over 1.5), jaundice, escalating ascites, and recurrent variceal bleeding;
- Hepatopulmonary, portopulmonary, and hepatorenal syndrome;
- Recurrent peritonitis;
- FEV1/FVC below 50% and severe malnutrition (contentious indication);
- Decline in quality of life [10,15,16,20,25,26,49].

Liver transplantation is contraindicated in the following circumstances:

- Presence of extrahepatic malignancies;
- Multiorgan disease;
- Severe pulmonary hypertension unresponsive to treatment;
- Pulmonary exacerbations;
- Hepatocarcinoma, CFRD, severe cardiopulmonary disease, colonization, or infections with multi-resistant bacteria (relative contraindications) [20].

Severity of liver disease alone is an insufficient criterion for the transplant decision; the lung disease stage must also be considered [41]. Eligibility is evaluated using the Pediatric End-Stage Liver Disease (PELD) score. Simultaneous liver and pancreas transplantation improves pancreas function and post-transplant body mass index [25].

The treatment protocol pre- and post-transplant includes the following:

- For patients at low risk of DIOS: administration of N-acetylcysteine, senna, electrolyte GI lavage solution, and nasogastric tube in those with delayed gastric emptying.
- For patients at high risk of DIOS: the same measures as those with low risk, with the addition of prophylactic loop ileostomy [20,47].

Post-transplant administration of CFTR modulators is controversial due to potential drug interactions. Each ETI combination component, like tacrolimus, is a CYP3A4/5 substrate. Monitoring liver transaminases and tacrolimus concentration is recommended after ETI initiation and then monthly [11].

G. Future Therapies

Investigations for potential future CF liver disease treatments include the following:

- Gene transfer via adenoviruses or liposomes;
- Chlorine channel agonists (purinogenic nucleotides);
- Colchicine, antioxidants, steroids, interferon, growth factor modulators to reduce fibrogenesis;
- Anti-inflammatory agents to mitigate the inflammatory response;
- Antiviral prophylaxis to diminish hepatocellular destruction;
- Glutathione to stimulate bile flow in hepatocytes;
- Src tyrosine kinases targeting TLR4-mediated inflammation to reduce pro-inflammatory state in human CF cholangiocytes;
- FGF19 analogues and other fibroblast growth factors, such as FGF1 (PPARγ target), with potential therapeutic efficacy;
- Vitamin D to inhibit liver fibrosis via the TGFβ1/SMAD signaling pathway;
- Antifibrotic substances (farnesoid X receptor agonist) acting at liver and intestinal levels, participating in bile acid homeostasis [18,24,40,98,99,100,101]. In the event of persistent symptoms despite treatment, it is advisable to reassess treatment adherence, consider altering the enzyme preparation, adjust the timing of administration, address gastric acidity, and conduct investigations to rule out other gastrointestinal disorders [102].

#### 3.3.2. Monitoring

The monitoring protocol for patients with CF and liver involvement comprises the following components:

- Annual consultations with gastroenterologists/hepatologists to evaluate cirrhosis severity, portal hypertension, and associated complications.
- Biochemical assessments (ALT, AST, GGT, ALP, prothrombin time, platelets, bilirubin) every 6 months; platelet monitoring is recommended, even if values do not suggest thrombocytopenia, due to early splenic sequestration in portal hypertension. Elevations exceeding 1.5 times normal values for transaminases, alkaline phosphatase, GGT, and bilirubin necessitate repeated analyses every 3–6 months and an abdominal ultrasound [8].
- Activation of abdominal ultrasound plus elastography annually, alternated with CT/MRI.
- Endoscopy every 2–3 years for individuals with cirrhosis or splenomegaly.
- Alpha-fetoprotein assessment every 6 months for those with cirrhosis.
- Semiannual screening for hepatocellular carcinoma.
- Liver function tests before initiating CFTR modulator therapy, followed by monthly assessments for the first year, and subsequently on an annual basis.
- Liver stiffness monitoring during CFTR modulator therapy for early detection of CFLD response or progression.
- Periodic monitoring of vitamins A, D, E, and prothrombin to prevent deficiencies or toxicity.

Oral Glucose Tolerance Test (OGTT) considerations, taking into account the increased incidence of CFRD observed in CF patients with liver heterogeneity or cirrhosis on ultrasounds [7,11,15,16,20,24,25,41,69].

### 3.4. Disease Progression and Prognosis

The presence of multiorgan dysfunction significantly amplifies morbidity, adversely impacting the overall quality of life [103]. In patients with CF, the coexistence of liver disease intricately complicates the clinical trajectory, resulting in shortened life expectancy, with a mortality rate ranging between 2.5 and 3.4% [2,15,25]. Variceal bleeding, liver transplantation, and liver-related mortality predominantly manifest within the initial decade following cirrhosis identification. Portal hypertension, a crucial factor in variceal bleeding-related mortality, is observed in 20–50% of cases within 10 years of diagnosis [97].

Symptoms of CFLD may remain inconspicuous or mild until the onset of portal hypertension. Consequently, progressive liver injury may correlate with deteriorating health before clinical manifestations of liver disease become evident [104]. Despite CFTR dysfunction affecting all patients, severe liver disease develops selectively, irrespective of genotype, implying the involvement of additional factors in its pathogenesis. Severe CFLD is considered life-threatening, primarily due to its adverse impact on respiratory function in conjunction with portal hypertension. Severe complications are infrequent in patients below 5 years of age but become notable in over 10% of those exceeding 30 years of age [2]. Hence, periodic screening for liver disease is imperative to detect hepatic damage in the pre-symptomatic phase through gastroenterological or hepatological consultations, biochemical assessments, and abdominal ultrasound or CT/MRI scans. Patients with cirrhosis warrant screenings for portal hypertension-related complications (esophageal/gastric varices, hepatopulmonary or portopulmonary syndrome) and liver failure [10]. Throughout the evolution of liver disease, transaminases, alkaline phosphatase, and GGT levels may exhibit fluctuations, often remaining within normal limits despite underlying histological changes [49].

Complications associated with cirrhosis and portal hypertension encompass malabsorption, fat-soluble vitamin deficiencies, gastrointestinal bleeding from varices (with a 6.7% risk according to Schwimmer et al. as cited by Leung et al. (2017), splenomegaly with hematological abnormalities, bacterial peritonitis, encephalopathy, and hepatorenal syndrome. Liver failure is an uncommon occurrence in CF-related cirrhosis [9,11,18,41,49].

Liver transplantation, either as a standalone procedure or in conjunction with lung transplantation, significantly enhances the disease’s course and prognosis. The survival rates post-transplantation stands at 89% at 1 year and 85.8% at 5 years, surpassing the 72% rate observed in adults [15,41].

The impact of early liver disease on health-related quality of life (HRQoL) in pediatric and adult CF patients remains less explored. To gauge the effect of early liver involvement in asymptomatic children with CF, several researchers have assessed generic and disease-specific HRQoL at the time of diagnosis and subsequently on an annual basis. These assessments aim to ascertain whether children with CF and their families undergo a decline in HRQoL amid the uncertainty surrounding the risk of liver disease progression. Notably, children exhibited relatively consistent HRQoL values, indicating a comparatively positive perception of health status, even across varying degrees of CFLD severity, including nodularity. However, HRQoL may not serve as a comprehensive tool for gauging the efficacy of early CFLD therapy [105,106].

## 4. Discussion

CFLD constitutes a diverse spectrum of hepatic manifestations in CF and stands out as one of the most prevalent nonpulmonary complications, alongside CFRD. Despite ongoing research efforts, the precise etiology of CFLD remains elusive, with the CFTR gene defect in CF being the widely acknowledged contributor. The escalating prevalence of CFLD underscores its multifactorial genesis. Establishing the age group most susceptible to CFLD development proves challenging, given the elevated transaminase levels in a significant percentage of CF patients within the initial two years of life, coupled with some remaining asymptomatic, only exhibiting elevated liver test values. The age-specific risk stratification for CFLD onset and incidence remains elusive due to its insidious initiation and the common occurrence of transient liver test abnormalities in children.

Timely identification and prompt initiation of treatment play pivotal roles in influencing both short-term and long-term prognoses. Despite endeavors in the specialized literature to delineate clear diagnostic criteria for CFLD, its heterogeneity necessitates individualized therapeutic approaches from the earliest stages. However, further investigations are imperative to establish a universally accepted definition of CFLD. Screening protocols, non-invasive technologies, and innovative biomarkers are indispensable for identifying patients at risk of cirrhosis or those manifesting silent signs of cirrhosis [26]. Recent studies involving FibroScan or shear wave elastography (SWE) in children and adolescents demonstrate promise, and the amalgamation of biochemical markers with either SWE or FibroScan exhibits potential for enhancing early CFLD identification [107,108].

CFLD serves as a confirmed risk factor for CFRD development. Furthermore, patients diagnosed with both CFLD and CFRD are at an increased susceptibility to experiencing a more severe hepatic involvement due to CF that can even progress to cirrhosis. Furthermore, findings from Colomba et al. (2019) underscore ALT as a marker of glucose abnormality in males with cystic fibrosis, prompting exploration into the interplay between liver disease phenotype, fibrosis risk, and the development of impaired glucose tolerance [109]. Concurrent CFLD and other complications, such as CFRD, necessitate personalized treatment approaches for optimal disease management and outcomes.

Early CFLD identification and timely intervention are crucial for averting complications in certain cases [11]. Given the advancements in CFTR-directed therapies and ongoing developments in liver-specific treatments, the identification of individuals with CF at high risk for CFLD becomes paramount. Exploring the intricate gut–liver axis may unveil novel therapeutic targets to impede the progression of chronic hepatobiliary diseases and enhance outcomes for children with CF. Future research avenues should delve into dietary manipulation, considering its controllable role in CFLD pathogenesis, to open new therapeutic perspectives, as no treatment has hitherto provided curative benefits for the disease. Gardiner et al. (2022) highlight the uncertainty surrounding whether CFTR modulators, within the era of precision medicine, can alter the risk of disease progression [110]. Recognizing that CF is more than just a lung disease and understanding that modulator therapies may influence the progression of pulmonary disease necessitates prospective clinical studies to examine the impact of novel CFTR modulators on various CF-related complications, including CFLD. Barriers such as advanced liver disease, adverse hepatic events, and economic constraints impede the equitable availability of CFTR modulators, demanding a comprehensive understanding of their safety profiles to identify patients at the highest risk of serious adverse events and achieve optimal risk–benefit ratios [69].

The evaluation of health-related quality of life constitutes a nuanced and inherently subjective instrument that furnishes valuable insights into the patient’s subjective assessment of their overall health. The quantification of health-related quality of life serves as a crucial resource, offering essential information for the judicious adaptation of care plans for these patients, as articulated by Jardine et al. and Williet et al. since 2014 [111,112].

## 5. Conclusions

CFLD, along with other complications of CF, poses a challenge wherein early diagnosis and intervention are imperative to impact both short-term and long-term outcomes. The validation of novel diagnostic methodologies and measures of therapeutic efficacy necessitates a consensus on CFLD definitions. Systematic and routine screening for liver impairment is essential to enable early detection and mitigate the progression of severe damage. UDCA, currently the sole therapy for CFLD patients, has not conclusively demonstrated an alteration in the natural course of the disease, and its early administration in recent years has not affected the incidence of severe forms of liver damage. The identification of perturbed elements within the gut–liver axis in CFLD may present novel opportunities for intervention using microbiome-based approaches such as bacteriophages, fecal microbial transplantation, and synthetic live bacterial therapeutics in this severe complication of CF. While drugs directly targeting CFTR protein dysfunction exhibit promising results, long-term studies are imperative to corroborate their effects.

## Figures and Tables

**Table 1:** Lesion scale assessment in liver elastography by Wiecek et al. (2022) [65].

Stages of Liver Fibrosis	METAVIR Score	Stiffness (kPa)
No fibrosis or portal fibrosis without septa	F0–F1	<3.95
Portal fibrosis with a few septa	F2	≤3.95, <7.0
Septal fibrosis with many septa but no cirrhosis	F3	≤7.0, <9.7
Cirrhosis	F4	≥9.7

**Table 2:** The effects of UDCA in CFLD.

Authors	Year	Type of the Study	Aim	Results
Colombo et al. [75]	1992	Dose– response	To establish whether improved efficacy could be obtained with higher doses	A significant improvement in transaminases with higher doses Similar improvement with the dose of 20 mg/kg body wt/day to that reported for patients with other liver diseases treated with lower doses
Mujtaba et al. [76]	2011	Retrospective	To evaluate effectiveness and safety of UDCA in CFLD	Improvement of LTs during treatment with UDCA No side effects No intolerance of UDCA
Ciucă et al. [28]	2015	Prospective	To evaluate the efficiency of UDCA on CFLD evolution	UDCA had beneficial effects on liver biochemical parameters (transaminases decreased) US score remained relatively constant, exception—severe cases (without impact)
Colombo et al. [77]	2016	Observational	To evaluate the fasting and postprandial serum bile acid composition in patients with CFLD after chronic administration of UDCA	Enhanced biotransformation of UDCA to the hepatotoxic secondary bile acid lithocholic acid occurs when the patients are treated with high doses
van derFeen et al. [78]	2016	Observational	To measure liver stiffness in CF patients treated with UDCA and in those without UDCA	UDCA reduced liver stiffness in patients with mild liver disease
Cheng et al. [79]	2017	Randomized controlled trials	To analyze if UDCA improves indices of liver function, reduces the risk of developing chronic liver disease, improves outcomes in CF	Improvement in the biliary excretion No significant changes after treatment with UDCA Not enough information about use in the long term More research on UDCA are needed
Colombo et al. [80]	2022	Retrospective multicenter cohort	To evaluate the incidence of severe liver disease in patients who received UDCA compared with those who do not receive it	Did not show a lower incidence of portal hypertension in CF patients treated with UDCA

**Table 3:** The effects of CFTR modulators in CFLD.

Author	Year	CFTR Modulator	Type of Study	Aim	Results
Wainwright et al. [83]	2015	LUMA/IVA	Double-blind, placebo-controlled, phase 3, randomized	To evaluate the incidence of adverse events in children ≥ 12 years old with CF	5.2% cases: elevation of ALT, AST > 3 × ULN
Davies et al. [84]	2016	IVA	Open-label, single-arm	To assess the safety, pharmacokinetics, and pharmacodynamics in children 2–5 years old	15%: LTs > 8 × ULN The only serious adverse event: raised concentrations of transaminases Monitoring should be frequent
Rowe et al. [85]	2017	IVA IVA/TEZ	Double-blind, placebo-controlled, phase 3, randomized	To evaluate the efficacy and safety in CF heterozygous, Phe508 del mutation	No clinically adverse events in the level of ALT, AST, total bilirubin
Heijerman et al. [86]	2019	IVA/TEZ ETI	Double-blind, randomized, phase 3	To evaluate the safety	Elevated transaminases level (2% IVA/TEZ; 4% ETI) No elevation in ALT/AST > 3 × ULN No elevation in bilirubin 2 × ULN No drug interruption/discontinuation of modulators
Kutney et al. [87]	2019	LUMA/IVA	Cross-sectional	To explore the impact of LUMA/IVA on hepatic steatosis in patients 11.3–39 years old	LUMA/IVA therapy is associated with reduced hepatic steatosis CFTR modulators should be included in future studies of hepatic steatosis/CFLD
van de Peppel et al. [88]	2019	IVA	Observational	To assess the effect of IVA on the enterohepatic circulation by assessing markers of bile acids homeostasis	IVA partially restored this disruption of bile acids homeostasis
Paluck et al. [89]	2021	LUMA/IVA	Retrospective chart review	To assess whether F508 del homozygous CF patients ≥ 12 years old have a derangement of LTs	Significant decrease in ALT, GGT, total bilirubin levels and no change in AST after 3 months of treatment
Drummond et al. [90]	2022	LUMA/IVA	Observational	Effects on features of liver involvement in F508 del homozygous CF adolescents	No hepatic adverse reactions No patient developed liver failure Serum levels of AST/ALT, GGT decreased A significant increase in biomarkers of CFTR activity
Levite et al. [91]	2023	LUMA/IVA	Retrospective	To observe alterations in LTs and fibrosis index	Decreases in GGT, APRI, GGT/platelets Improves liver fibrosis
		ETI			No differences
Kennedy et al. [92]	2023	ETI	Retrospective observational	To evaluate LTs	16%: elevated LTs Only 2.5% LTs > 5 × ULN Lower severity of derangements
				To evaluate the aspect of the liver by ultrasound	No patients with significant changes on liver US
				To evaluate the safety	CFTR modulators are safe

Abbreviations: LUMA/IVA = lumacaftor/ivacaftor; IVA = ivacaftor; IVA/TEZ = ivacaftor/tezacaftor; ETI = elexacaftor/tezacaftor/ivacaftor; LT = liver tests; ULN = upper limit of normal; AST = aspartate aminotransferase; ALT = alanine aminotransferase; GGT = gamma-glutamyl transferase.
